# AlphaFold Protein Structure Database in 2024: providing structure coverage for over 214 million protein sequences

**DOI:** 10.1093/nar/gkad1011

**Published:** 2023-11-02

**Authors:** Mihaly Varadi, Damian Bertoni, Paulyna Magana, Urmila Paramval, Ivanna Pidruchna, Malarvizhi Radhakrishnan, Maxim Tsenkov, Sreenath Nair, Milot Mirdita, Jingi Yeo, Oleg Kovalevskiy, Kathryn Tunyasuvunakool, Agata Laydon, Augustin Žídek, Hamish Tomlinson, Dhavanthi Hariharan, Josh Abrahamson, Tim Green, John Jumper, Ewan Birney, Martin Steinegger, Demis Hassabis, Sameer Velankar

**Affiliations:** European Molecular Biology Laboratory, European Bioinformatics Institute, Hinxton, UK; European Molecular Biology Laboratory, European Bioinformatics Institute, Hinxton, UK; European Molecular Biology Laboratory, European Bioinformatics Institute, Hinxton, UK; European Molecular Biology Laboratory, European Bioinformatics Institute, Hinxton, UK; European Molecular Biology Laboratory, European Bioinformatics Institute, Hinxton, UK; European Molecular Biology Laboratory, European Bioinformatics Institute, Hinxton, UK; European Molecular Biology Laboratory, European Bioinformatics Institute, Hinxton, UK; European Molecular Biology Laboratory, European Bioinformatics Institute, Hinxton, UK; School of Biological Sciences, Seoul National University, Seoul, South Korea; School of Biological Sciences, Seoul National University, Seoul, South Korea; Google DeepMind, London, UK; Google DeepMind, London, UK; Google DeepMind, London, UK; Google DeepMind, London, UK; Google DeepMind, London, UK; Google DeepMind, London, UK; Google DeepMind, London, UK; Google DeepMind, London, UK; Google DeepMind, London, UK; European Molecular Biology Laboratory, European Bioinformatics Institute, Hinxton, UK; School of Biological Sciences, Seoul National University, Seoul, South Korea; Google DeepMind, London, UK; European Molecular Biology Laboratory, European Bioinformatics Institute, Hinxton, UK

## Abstract

The AlphaFold Database Protein Structure Database (AlphaFold DB, https://alphafold.ebi.ac.uk) has significantly impacted structural biology by amassing over 214 million predicted protein structures, expanding from the initial 300k structures released in 2021. Enabled by the groundbreaking AlphaFold2 artificial intelligence (AI) system, the predictions archived in AlphaFold DB have been integrated into primary data resources such as PDB, UniProt, Ensembl, InterPro and MobiDB. Our manuscript details subsequent enhancements in data archiving, covering successive releases encompassing model organisms, global health proteomes, Swiss-Prot integration, and a host of curated protein datasets. We detail the data access mechanisms of AlphaFold DB, from direct file access via FTP to advanced queries using Google Cloud Public Datasets and the programmatic access endpoints of the database. We also discuss the improvements and services added since its initial release, including enhancements to the Predicted Aligned Error viewer, customisation options for the 3D viewer, and improvements in the search engine of AlphaFold DB.

## Graphical Abstract

**Graphical Abstract:**
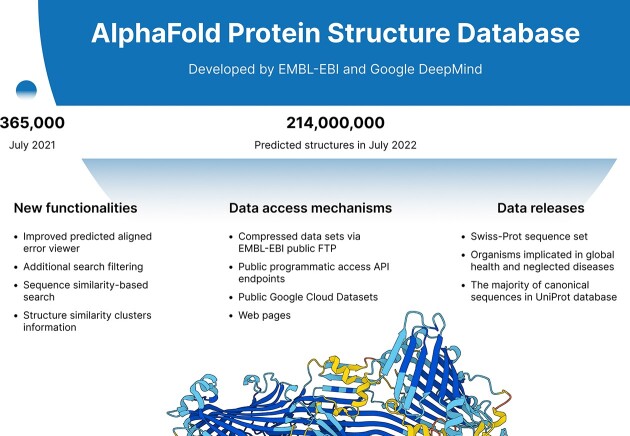

## Introduction

In the past few years, the landscape of protein structure prediction has evolved significantly due to the advent of next-generation tools such as AlphaFold (1), RoseTTAFold (2), and OpenFold (3), among others (4). The development of these tools was enabled by decades of research in protein sequences and structures and underscored the importance of open data and fundamental data resources like the Protein Data Bank (PDB) (5) and the Universal Protein Resource (UniProt) (6).

The new generation of predicted protein structure models has demonstrated remarkable accuracy, which could mitigate the continually widening gap between known protein sequences and experimentally determined protein structures (7). Accessing accurate protein structures paves the way for an enhanced understanding of protein function, providing researchers with the tools necessary for modulating these proteins or engineering new ones (8–10). As a result, numerous areas within the life sciences have experienced considerable impact due to the availability of accurately predicted protein structures. The primary domains notably affected include structure determination, structure-based drug discovery, and structural bioinformatics (11–13).

In the wake of the latest breakthroughs in prediction software, new databases such as the AlphaFold Protein Structure Database and the ESM Atlas (14) have emerged. With the availability of performant prediction software, a pertinent question arises: Why is there a need for databases for predicted structures when researchers can run the software on their proteins of interest?

While many new-generation protein structure predictors are readily accessible for the scientific community to run on arbitrary input protein sequences, a significant barrier exists for researchers less acquainted with using scientific software. Moreover, in large-scale bioinformatics analysis like comparative or functional analysis, the necessity to predict a massive number of protein structures becomes computationally expensive and redundant, contributing to a significant carbon footprint. Even in the case of single protein predictions, looking up pre-generated structures takes seconds compared to potentially hours of computation time. The absence of pre-generated structures also obstructs the integration of these valuable predictions into core data resources like UniProt (6), InterPro (15), Ensembl (16) or the PDBe—Knowledge Base (17).

**Figure 1.**
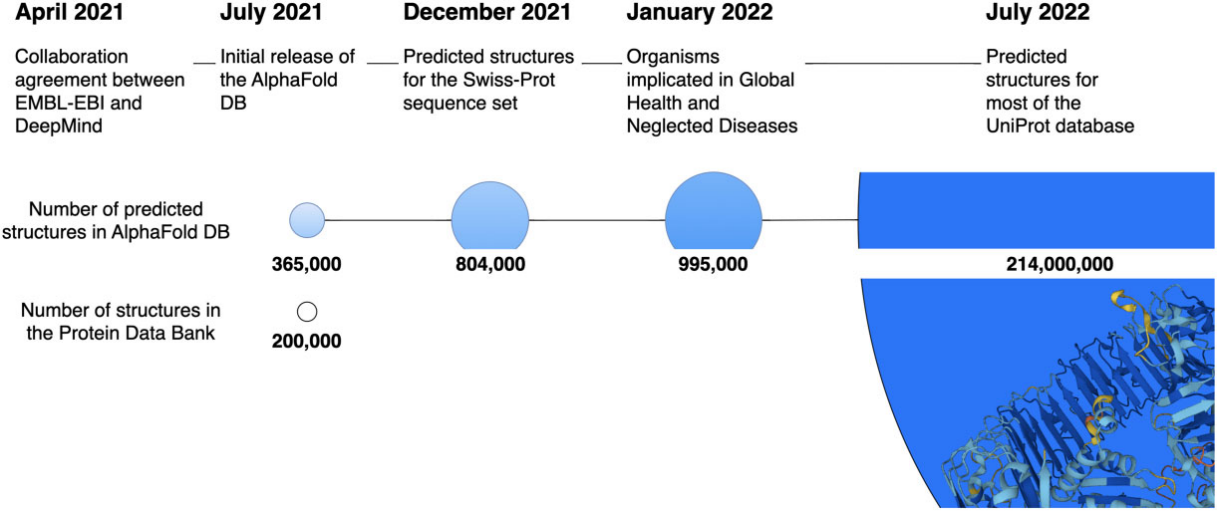
The expansion of AlphaFold DB. The AlphaFold Protein Structure Database increased in size through consecutive releases. As of September 2023, it archives over 214 million predicted protein structures.

This paper presents the data updates and functionality improvements in the AlphaFold Protein Structure Database, a collaborative project between EMBL-EBI and Google DeepMind, since its initial launch in July 2021. We outline how the coverage of sequence space has improved from the initial release, with the structure count increasing from approximately 300k to over 214 million (Figure 1). Additionally, we provide insights into the changes and additions to the metadata and the format of confidence metrics such as the Predicted Aligned Error (PAE). Updates on accessing the data and the data types available through File Transfer Protocol (FTP), our Application Programming Interface (API), and bulk download options will be discussed. Lastly, we give an overview of all the improvements and new functionalities introduced on the AlphaFold DB website and give an overview of possible future directions.

## Implementation

Updating the AlphaFold DB is a multifaceted process, encompassing multiple stages of data management. This process includes generating a vast array of protein structure predictions, organising these predictions in a structured and searchable format, and ensuring straightforward and efficient data access for users. The ultimate result of this process is a comprehensive and user-oriented experience, facilitating cutting-edge research across diverse fields within the life sciences.

### Data generation

The data generation process for the AlphaFold DB is carried out by Google DeepMind, with all predictions stored in PDB, mmCIF and binaryCIF formats, along with their corresponding metadata in JSON format. The generated mmCIF files adhere to the modelCIF format (18).

AlphaFold yields a per-residue estimate of its confidence, known as pLDDT, ranging from 0 to 100, indicating the tool's predicted score on the lDDT-Cα metric (19). The residue-wise pLDDT scores are stored in the B-factor fields of PDB files and under the *‘_ma_qa_metric_local’* category in mmCIF files. Regions with pLDDT greater than 90 are generally modelled with high accuracy, making them suitable for high accuracy-dependent applications such as characterising binding sites. Those with pLDDT between 70 and 90 are generally well-modelled, representing a reliable backbone prediction. On the other hand, regions with pLDDT between 50 and 70 have lower confidence and should be used cautiously. Finally, regions with pLDDT scores below 50 often exhibit a spaghetti-like appearance in 3D view, indicating probable disordered regions. Structured domains with many inter-residue contacts are generally more reliable than extended linkers or isolated long helices. Unphysical bond lengths and clashes usually do not appear in the confident structured regions, and any region with several of these should be disregarded. Regardless of the absolute pLDDT score, the PDB and mmCIF files provide coordinates for all regions, and it is incumbent upon the user to interpret the model prudently, in line with the provided guidance.

In addition to the predicted atomic coordinates and the pLDDT scores, AlphaFold generates a ‘Predicted Aligned Error’ (PAE) output, representing the predicted error between relative positions of residue pairs. PAE is a measure indicating confidence in the relative positions of larger structural units of proteins, like domains. The PAE can be used to assess the spatial location of protein domains by looking at the values for residue pairs between different domains. The raw data with PAE for all residue pairs can be downloaded as a JSON file. However, parsing the JSON file requires Python or another programming language for analysis or visualisation.

In 2022, the JSON file format was updated to a more compact representation. It now consists of a *‘predicted_aligned_error’* field instead of the 1D *‘distances’* field in the earlier representation and a ‘*max_predicted_aligned_error*’ field indicating the maximum possible value of PAE.

### Data archiving

Data archiving for AlphaFold DB began with an initial release in July 2021, housing over 360 000 structures for 20 model organism proteomes with sequences derived from the ‘one sequence per gene’ reference proteomes provided in UniProt release 2021_02. In December 2021, most of the reviewed sequences in UniProt, i.e. the Swiss-Prot dataset, were incorporated from the UniProt release 2021_04. In January 2022, proteomes relevant to global health, derived from priority lists by the World Health Organization, were added, utilising sequences from UniProt release 2021_04 ‘one sequence per gene’ reference proteomes. By July 2022, most of the remaining sequences from UniProt release 2021_04 were included, featuring an additional TAR file on the AFDB download page, EMBL-EBI’s FTP and Google Cloud Datasets, containing predictions in MANE select (20).

A November 2022 update rectified structures affected by a temporary numerical bug presented in the July release. This bug led to low accuracy predictions with correspondingly low pLDDT for ∼4% of the total structure predictions in the database. As part of this update, the coordinates for affected structures were updated (old coordinate files remain accessible as v3 files), and minor metadata adjustments were made in the mmCIF files for the remaining structures. We document every data version update in our changelog at https://ftp.ebi.ac.uk/pub/databases/alphafold/CHANGELOG.txt.

As of September 2023, the EMBL-EBI’s FTP area hosts TAR files for proteomes of 48 organisms, including model organisms and WHO pathogens of interest (Supplementary Table S1). The complete dataset is stored on Google Cloud Platform (GCP) and is accessed through a file access API. The metadata is indexed using Apache-Solr powering search API to facilitate data accessibility and searchability.

The database provides access to over 214 million predicted structures, although some sequences might be outdated compared to UniProt due to less frequent data releases in the AlphaFold DB. Predictions of UniProt sequences are outputs of a single model run. In contrast, Swiss-Prot/proteomes entries represent the most confident prediction from runs of five models trained with different random seeds. The following sequences are not covered in the database: (i) those that are less than 16 amino acids, or (ii) >2700 for SwissProt or proteome sequences and 1280 for other UniProt sequences, or (iii) those that contain non-standard amino acids, or (iv) are not in the UniProt ‘one sequence per gene’ FASTA file, or (v) viral proteins. These limitations are under discussion.

### Data access

We facilitate access to the predicted structures and their associated confidence metrics from AlphaFold DB through four different channels: FTP, Google Cloud Public Data, API, and directly from the AlphaFold DB web page.

A subset of the AlphaFold DB can be accessed through EMBL-EBI’s public FTP area at http://ftp.ebi.ac.uk/pub/databases/alphafold/. The FTP area houses a comprehensive README.txt file containing detailed information about all available files. Predictions are archived according to versions, with all versions, including the latest release, being accessible from folders within the FTP area. For example, the latest archived files are available via http://ftp.ebi.ac.uk/pub/databases/alphafold/latest/. Additionally, supplementary files such as sequences in FASTA format for all the predictions, a CSV file listing UniProt accessions with predicted structures and a CHANGELOG file highlighting version control are provided to help users. It is important to note that PAE data is unavailable from the EMBL-EBI public FTP.

The full dataset, housing all predictions, is accessible from Google Cloud Public Datasets under a CC-BY-4.0 license. This dataset, approximately 23 TiB in size, is available at the following Google Cloud Storage Bucket: gs://public-datasets-deepmind-alphafold-v4. We suggest that most users download only the subset of files relevant to their specific use case to optimise resources. However, if a complete dataset is required for local processing, as might be the case in an academic high-performance computing centre, it can be downloaded in roughly 2.5 days using a 1 Gbps internet connection. Importantly, a Google account is necessary for the download.

The Alphafold DB API provides an efficient way for developers to programmatically access metadata associated with all archived AlphaFold predictions. The API facilitates information retrieval related to protein structures predicted by AlphaFold, such as URLs of model files (mmCIF, binaryCIF and PDB), model quality metrics, and other valuable information. All available API endpoints are keyed on UniProt accessions, and an interactive API documentation can be found at https://www.alphafold.ebi.ac.uk/api-docs.

**Figure 2.**
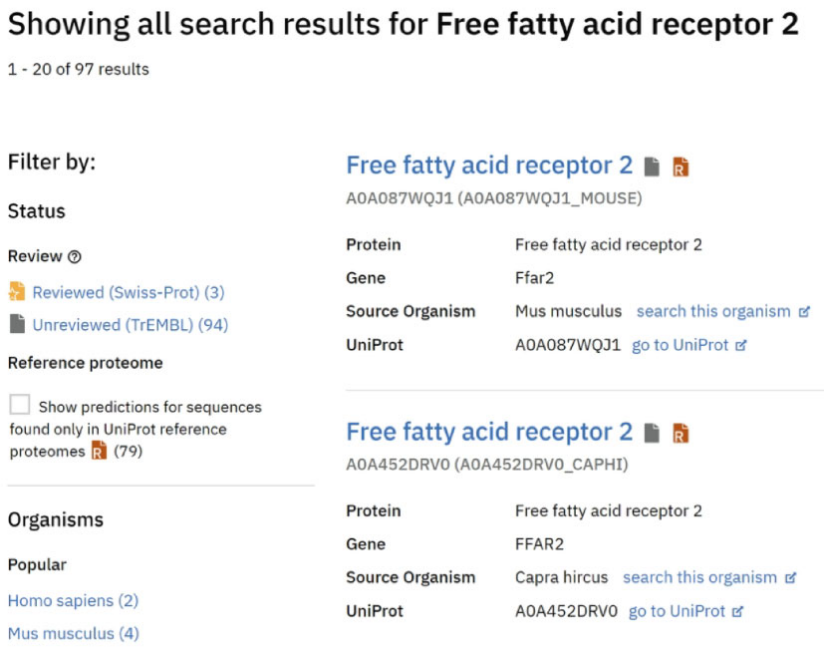
Improve search results UI. The improved search UI includes more filtering options based on the underlying sequence of the protein structures and easier access to the most popular organisms.

Finally, structure predictions can be directly accessed through the AlphaFold DB website. The interface offers an intuitive search functionality to quickly find and download protein structure predictions and their corresponding confidence metrics. Our commitment to enhancing user experience and removing ambiguity has resulted in several improvements to our user interface (UI) since its initial launch. One notable advancement is the refined UI for search results, ensuring an intuitive and user-friendly experience (Figure 2).

We have added filtering functionality to provide users with a more tailored browsing experience. Users can now filter search results based on the status of the underlying sequence. This feature allows users to narrow their results to only reviewed (Swiss-Prot) or unreviewed (TrEMBL) UniProt accessions, enabling them to focus on predicted structures derived from higher-quality, curated protein sequences.

An additional new option allows users to filter results based on whether the protein is part of a UniProt reference proteome dataset. This filter offers an additional indicator of confidence in the quality of the sequence. Recognising the popularity of specific organisms, we have also created a distinct list featuring the most frequently searched-for species, allowing users to swiftly find structures associated with popular species, like Human, Mouse or *Escherichia coli*, enhancing our platform's overall user-friendliness and efficiency.

In response to frequent requests from the user community since the AlphaFold DB launch, we have made significant strides to implement a sequence-based similarity search feature. We have incorporated the Basic Local Alignment Search Tool (BLAST) (21) into our Google Cloud Platform infrastructure to achieve this. This implementation allows our system to swiftly compare user-provided protein sequences against our database. We developed an API to send user protein sequences to the BLAST service and retrieve the responses. To integrate these new search results into our internal search engine, we built on the XJoin functionality in the BioSolr plugin (https://github.com/flaxsearch/BioSolr), further extending it to suit our specific requirements. This functionality ensures seamless integration of the BLAST search results and facilitates support for conventional filtering options.

**Figure 3.**
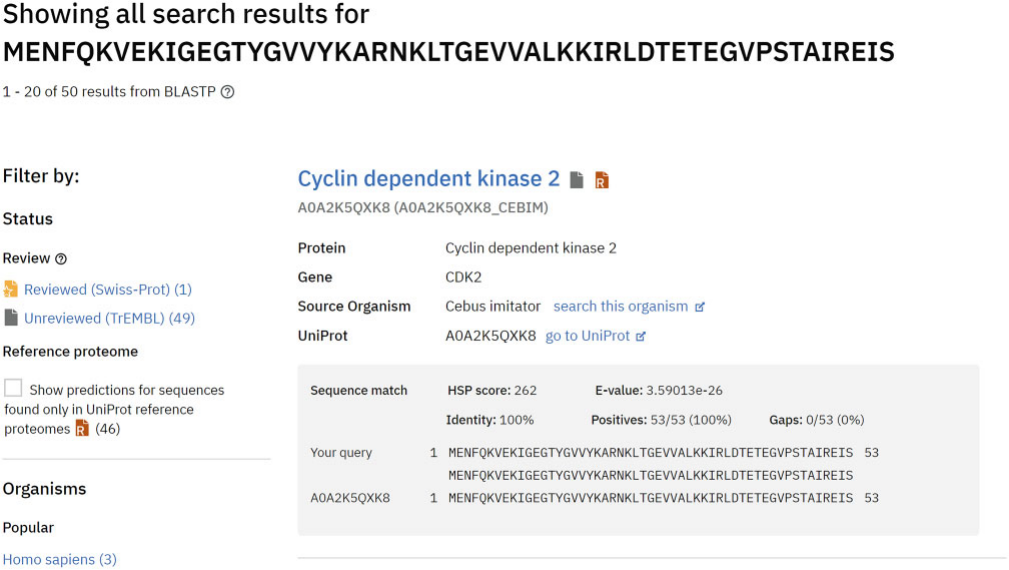
Sequence similarity search results. We added support for performing sequence similarity searches in AlphaFold DB. The search results page displays a list of predicted structures with sequences similar to the user's.

We provide an intuitive user interface to present the sequence search results clearly and comprehensively (Figure 3). The dedicated results page lists all similar proteins for which we have predicted structures, and it offers capabilities for both sorting and filtering, enhancing the ability to navigate the results efficiently.

**Figure 4.**
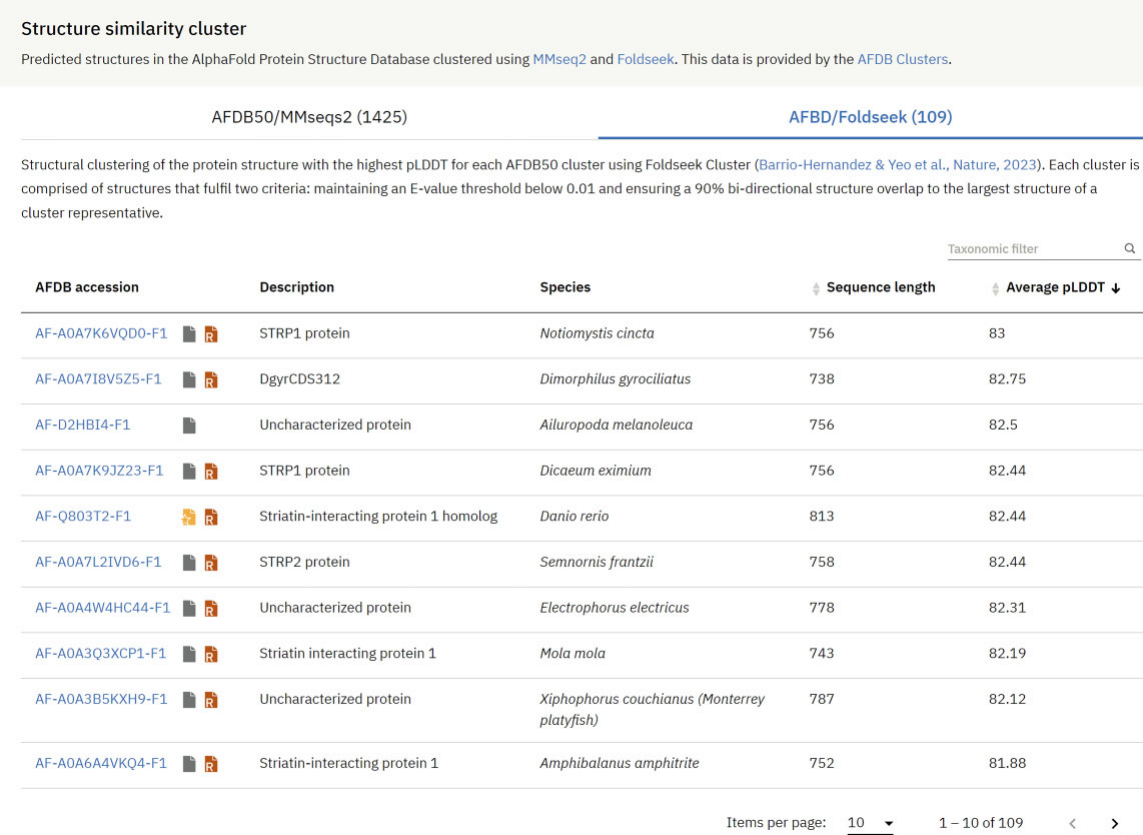
Structure similarity cluster members. Using data from AFDB Clusters, we display lists of AlphaFold predictions structurally similar to a protein of interest.

With over 214 million predicted protein structures, the AlphaFold DB presents a colossal challenge in data analysis. Tackling this challenge, Steinegger et al. recently unveiled the Foldseek Cluster, a state-of-the-art structural-alignment-based clustering algorithm designed for vast datasets (22). They applied this novel method to the AlphaFold DB archive, making the derived structure similarity clusters available to the research community. We integrated these structure similarity clusters and continue to work on deploying a structure-based similarity search into the AlphaFold DB. As part of the initial rollout in our phased-release approach, tables have been incorporated into the AFDB prediction pages, listing AlphaFold predictions from the same similarity cluster as the protein of interest (Figure 4). The clustering process was two-pronged: the MMseqs2 tool first clustered 214 million UniProtKB protein sequences from AlphaFold DB, trimming it down to 52 million clusters based on defined sequence criteria. A protein with the peak pLDDT score was elected as each cluster's representative. This set then underwent a secondary clustering via Foldseek, using specific structural delineations, resulting in 18.8 million clusters. After dismissing sequences recognised as fragments, we finalised 2.30 million robust clusters, each housing at least a pair of structures. We provide access to AlphaFold predictions from the two main categories generated by the clustering process: AFDB/Foldseek and AFDB50/MMseqs. These results are also accessible through the public API of AlphaFold DB.

The Predicted Aligned Error (PAE) plays a pivotal role as a confidence metric to evaluate protein structures predicted by AlphaFold. Since the initial launch of AlphaFold DB, we have incorporated an interactive 2D heatmap visualisation on the prediction pages. This visualisation tool allows users to focus on specific regions and assess the confidence of AlphaFold's prediction regarding the regions’ relative positioning.

**Figure 5.**
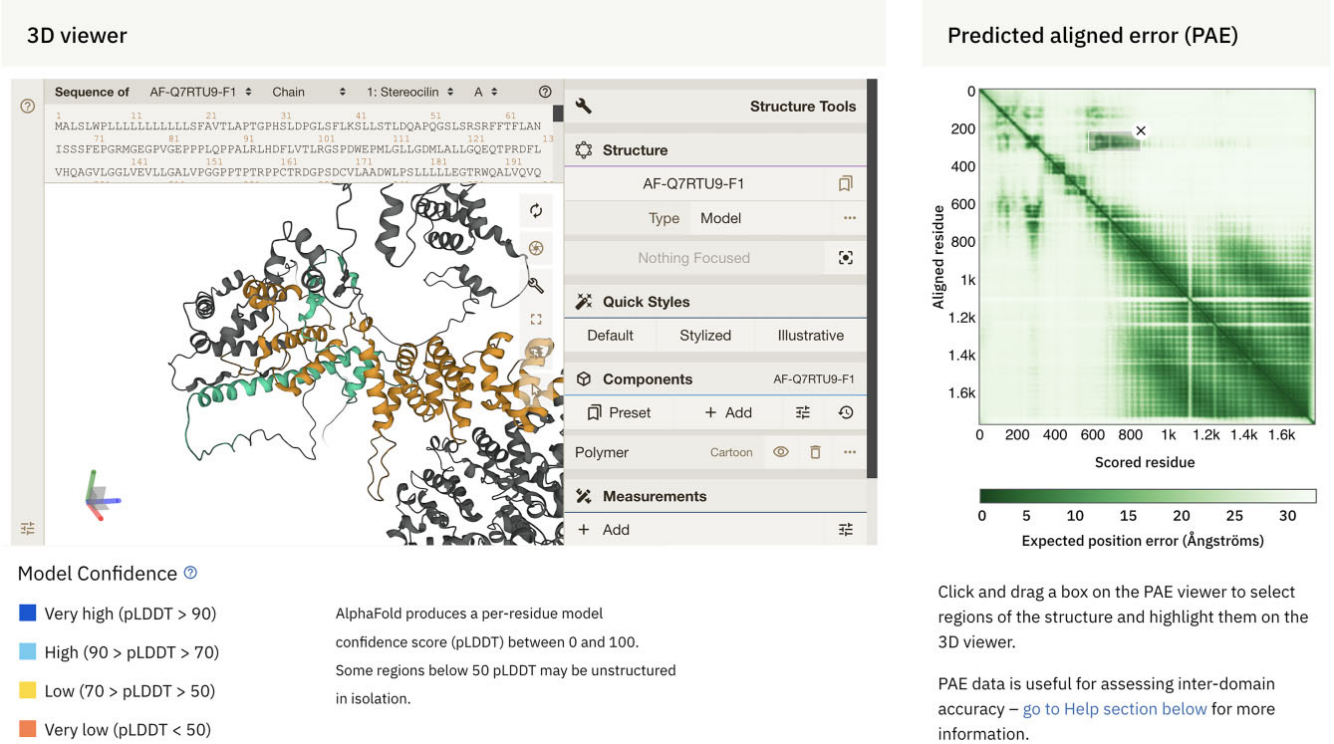
Improved support for highlighting non-consecutive regions. The new version of the interactive PAE viewer makes it easier to distinguish between highlighted non-consecutive regions when assessing their relative positions' confidence, as shown for AlphaFold DB accession https://alphafold.ebi.ac.uk/entry/Q7RTU9.

Understanding the importance of PAE in providing users insights into the relative orientation of protein domains, we have taken measures to enhance this feature further. We improved the visualisation of non-consecutive regions and the interactivity between the PAE viewer and the 3D molecular graphics viewer, Mol* (23) (Figure 5). Now, when users select regions on the off-diagonal segment of the PAE heatmap, the corresponding regions in the 3D view are highlighted. This improvement not only bolsters the accessibility of the PAE data but also empowers users to make informed interpretations concerning the overall conformation of the predicted structure.

**Figure 6.**
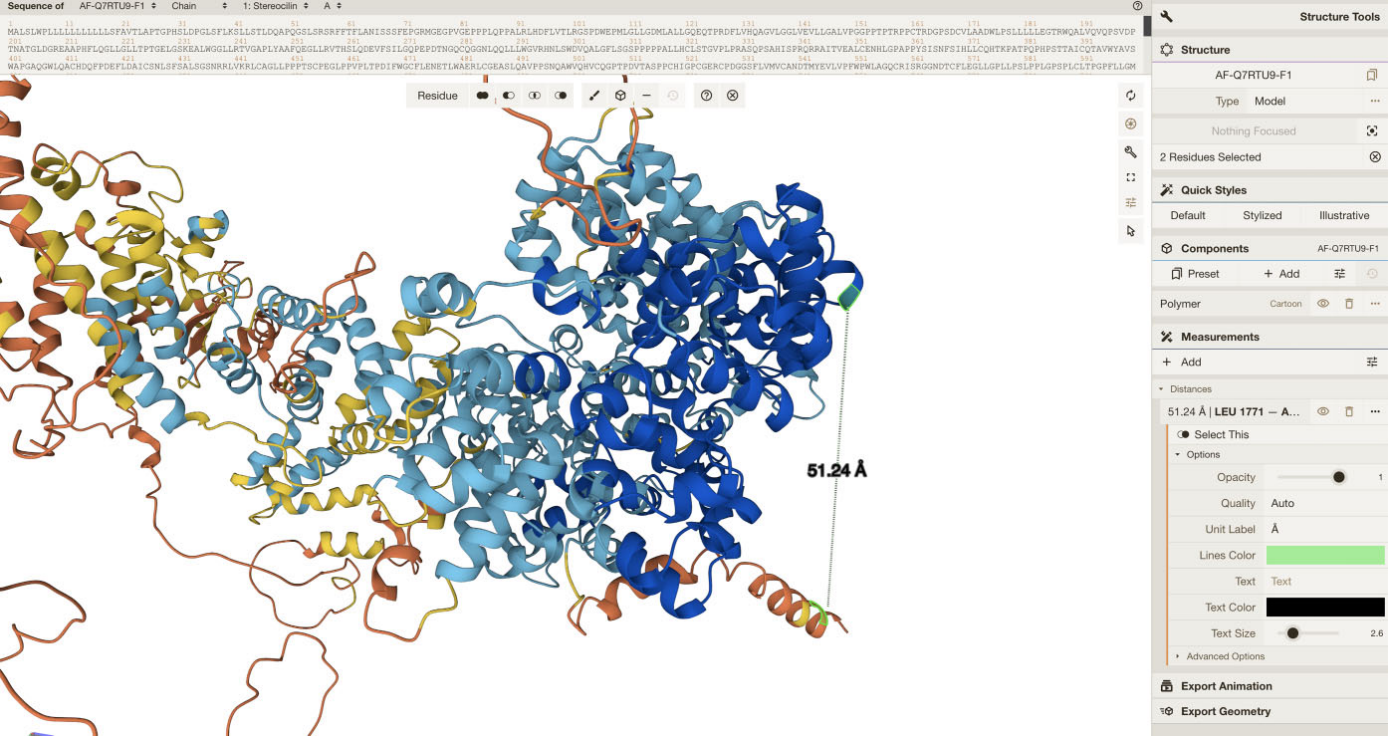
Improved customisation in Mol*. Enhanced customisation options in Mol* allow users to perform popular actions such as measuring distances or changing the rendering style.

In addition to improving the interaction between Mol* and the PAE viewer, we further expanded the capabilities of our 3D molecular viewer to cater to more advanced analysis in response to user feedback. Now, users can select individual atoms, residues, and complete chains, facilitating a more comprehensive and focused exploration of molecular structures. A practical application of this functionality is measuring the distances between residue pairs, a frequent action of in-depth investigation of protein structures (Figure 6).

## Conclusions and outlook

Accessing hundreds of millions of predicted protein structures housed within data resources such as the AlphaFold Protein Structure Database marks a significant leap for structural biology and has impacted diverse fields within the life sciences. The impact of the predicted structures is further enhanced by their seamless integration into several core data resources, including PDBe-KB (17), UniProt (6), Ensembl (16), InterPro (15), Genecards (24) and MobiDB (25), among others. However, while the field has made significant strides, there are discernible gaps in both data representation and functionality that we are committed to addressing.

There are intriguing frontiers for data enhancement, including adding structures for isoforms and targeted datasets for multimeric predicted protein structures. In parallel with these anticipated data updates, we are also working towards enriching the predicted structures with domain annotations (26), integrating small molecules through AlphaFill (27), referencing cross-linking data, and devising dedicated pages tailored for fragments. These planned improvements are based on our ongoing interactions with the scientific community, whose feedback and insights highlight areas where the most impactful changes could be made. We invite all users to share their suggestions through the AlphaFold DB helpdesk (afdbhelp@ebi.ac.uk).

While the availability of millions of predicted protein structures promises valuable insights into molecular biology, they might be behind a barrier for many researchers who may lack familiarity with handling macromolecular structure data and may not sufficiently understand the strengths and limitations inherent in predicted structures. To address these challenges and make protein structure data more accessible, we now focus on providing a comprehensive training platform, enabling the broader scientific community to use structural data more efficiently.

Arguably, we have entered a new era in structural biology, when the abundance of available predicted protein structure data enables researchers to probe an unprecedented range of biological questions. As stewards of AlphaFold DB, we are committed to bolstering its accessibility, hoping to amplify its transformative impact on science and society.

## Supplementary Material

gkad1011_Supplemental_File

## Data Availability

Versioned TAR files for 48 model organisms and pathogens and metadata files are available from the public FTP area of EMBL-EBI at http://ftp.ebi.ac.uk/pub/databases/alphafold/. The documentation of the public API endpoints of AlphaFold DB is available at https://www.alphafold.ebi.ac.uk/api-docs. A guide on accessing all the Google Cloud Public Dataset data is available from our download page https://www.alphafold.ebi.ac.uk/download.
