# Supplementary material for: AlphaFold Protein Structure Database in 2024: providing structure coverage for over 214 million protein sequences
Source: Nucleic Acids Res. 2023 Nov 2;52(D1):D368–75. doi: 10.1093/nar/gkad1011 (PMC10767828; doi:10.1093/nar/gkad1011)
Supplement: gkad1011_Supplemental_File [file gkad1011_supplemental_file.docx]

| **Species** | **Reference proteome** | **Predicted structures** | **Download size** |
| --- | --- | --- | --- |
| *Ajellomyces capsulatus* | [UP000001631](https://www.uniprot.org/proteomes/UP000001631) | 9,199 | [1,366 MB](https://ftp.ebi.ac.uk/pub/databases/alphafold/latest/UP000001631_447093_AJECG_v4.tar) |
| *Arabidopsis thaliana* | [UP000006548](https://www.uniprot.org/proteomes/UP000006548) | 27,434 | [3,719 MB](https://ftp.ebi.ac.uk/pub/databases/alphafold/latest/UP000006548_3702_ARATH_v4.tar) |
| *Brugia malayi* | [UP000006672](https://www.uniprot.org/proteomes/UP000006672) | 8,743 | [1,288 MB](https://ftp.ebi.ac.uk/pub/databases/alphafold/latest/UP000006672_6279_BRUMA_v4.tar) |
| *Caenorhabditis elegans* | [UP000001940](https://www.uniprot.org/proteomes/UP000001940) | 19,694 | [2,655 MB](https://ftp.ebi.ac.uk/pub/databases/alphafold/latest/UP000001940_6239_CAEEL_v4.tar) |
| *Campylobacter jejuni* | [UP000000799](https://www.uniprot.org/proteomes/UP000000799) | 1,620 | [175 MB](https://ftp.ebi.ac.uk/pub/databases/alphafold/latest/UP000000799_192222_CAMJE_v4.tar) |
| *Candida albicans* | [UP000000559](https://www.uniprot.org/proteomes/UP000000559) | 5,974 | [985 MB](https://ftp.ebi.ac.uk/pub/databases/alphafold/latest/UP000000559_237561_CANAL_v4.tar) |
| *Cladophialophora carrionii* | [UP000094526](https://www.uniprot.org/proteomes/UP000094526) | 11,170 | [1,734 MB](https://ftp.ebi.ac.uk/pub/databases/alphafold/latest/UP000094526_86049_9EURO1_v4.tar) |
| *Danio rerio* | [UP000000437](https://www.uniprot.org/proteomes/UP000000437) | 24,664 | [4,224 MB](https://ftp.ebi.ac.uk/pub/databases/alphafold/latest/UP000000437_7955_DANRE_v4.tar) |
| *Dictyostelium discoideum* | [UP000002195](https://www.uniprot.org/proteomes/UP000002195) | 12,622 | [2,193 MB](https://ftp.ebi.ac.uk/pub/databases/alphafold/latest/UP000002195_44689_DICDI_v4.tar) |
| *Dracunculus medinensis* | [UP000274756](https://www.uniprot.org/proteomes/UP000274756) | 10,834 | [1,366 MB](https://ftp.ebi.ac.uk/pub/databases/alphafold/latest/UP000274756_318479_DRAME_v4.tar) |
| *Drosophila melanogaster* | [UP000000803](https://www.uniprot.org/proteomes/UP000000803) | 13,458 | [2,218 MB](https://ftp.ebi.ac.uk/pub/databases/alphafold/latest/UP000000803_7227_DROME_v4.tar) |
| *Enterococcus faecium* | [UP000325664](https://www.uniprot.org/proteomes/UP000325664) | 2,823 | [289 MB](https://ftp.ebi.ac.uk/pub/databases/alphafold/latest/UP000325664_1352_ENTFC_v4.tar) |
| *Escherichia coli* | [UP000000625](https://www.uniprot.org/proteomes/UP000000625) | 4,363 | [458 MB](https://ftp.ebi.ac.uk/pub/databases/alphafold/latest/UP000000625_83333_ECOLI_v4.tar) |
| *Fonsecaea pedrosoi* | [UP000053029](https://www.uniprot.org/proteomes/UP000053029) | 12,509 | [2,020 MB](https://ftp.ebi.ac.uk/pub/databases/alphafold/latest/UP000053029_1442368_9EURO2_v4.tar) |
| *Glycine max* | [UP000008827](https://www.uniprot.org/proteomes/UP000008827) | 55,799 | [7,293 MB](https://ftp.ebi.ac.uk/pub/databases/alphafold/latest/UP000008827_3847_SOYBN_v4.tar) |
| *Haemophilus influenzae* | [UP000000579](https://www.uniprot.org/proteomes/UP000000579) | 1,662 | [176 MB](https://ftp.ebi.ac.uk/pub/databases/alphafold/latest/UP000000579_71421_HAEIN_v4.tar) |
| *Helicobacter pylori* | [UP000000429](https://www.uniprot.org/proteomes/UP000000429) | 1,538 | [167 MB](https://ftp.ebi.ac.uk/pub/databases/alphafold/latest/UP000000429_85962_HELPY_v4.tar) |
| *Homo sapiens* | [UP000005640](https://www.uniprot.org/proteomes/UP000005640) | 23,391 | [4,877 MB](https://ftp.ebi.ac.uk/pub/databases/alphafold/latest/UP000005640_9606_HUMAN_v4.tar) |
| *Klebsiella pneumoniae* | [UP000007841](https://www.uniprot.org/proteomes/UP000007841) | 5,727 | [561 MB](https://ftp.ebi.ac.uk/pub/databases/alphafold/latest/UP000007841_1125630_KLEPH_v4.tar) |
| *Leishmania infantum* | [UP000008153](https://www.uniprot.org/proteomes/UP000008153) | 7,924 | [1,511 MB](https://ftp.ebi.ac.uk/pub/databases/alphafold/latest/UP000008153_5671_LEIIN_v4.tar) |
| *Madurella mycetomatis* | [UP000078237](https://www.uniprot.org/proteomes/UP000078237) | 9,561 | [1,541 MB](https://ftp.ebi.ac.uk/pub/databases/alphafold/latest/UP000078237_100816_9PEZI1_v4.tar) |
| *Methanocaldococcus jannaschii* | [UP000000805](https://www.uniprot.org/proteomes/UP000000805) | 1,773 | [174 MB](https://ftp.ebi.ac.uk/pub/databases/alphafold/latest/UP000000805_243232_METJA_v4.tar) |
| *Mus musculus* | [UP000000589](https://www.uniprot.org/proteomes/UP000000589) | 21,615 | [3,619 MB](https://ftp.ebi.ac.uk/pub/databases/alphafold/latest/UP000000589_10090_MOUSE_v4.tar) |
| *Mycobacterium leprae* | [UP000000806](https://www.uniprot.org/proteomes/UP000000806) | 1,602 | [177 MB](https://ftp.ebi.ac.uk/pub/databases/alphafold/latest/UP000000806_272631_MYCLE_v4.tar) |
| *Mycobacterium tuberculosis* | [UP000001584](https://www.uniprot.org/proteomes/UP000001584) | 3,988 | [431 MB](https://ftp.ebi.ac.uk/pub/databases/alphafold/latest/UP000001584_83332_MYCTU_v4.tar) |
| *Mycobacterium ulcerans* | [UP000020681](https://www.uniprot.org/proteomes/UP000020681) | 9,033 | [584 MB](https://ftp.ebi.ac.uk/pub/databases/alphafold/latest/UP000020681_1299332_MYCUL_v4.tar) |
| *Neisseria gonorrhoeae* | [UP000000535](https://www.uniprot.org/proteomes/UP000000535) | 2,106 | [196 MB](https://ftp.ebi.ac.uk/pub/databases/alphafold/latest/UP000000535_242231_NEIG1_v4.tar) |
| *Nocardia brasiliensis* | [UP000006304](https://www.uniprot.org/proteomes/UP000006304) | 8,372 | [874 MB](https://ftp.ebi.ac.uk/pub/databases/alphafold/latest/UP000006304_1133849_9NOCA1_v4.tar) |
| *Onchocerca volvulus* | [UP000024404](https://www.uniprot.org/proteomes/UP000024404) | 12,047 | [1,624 MB](https://ftp.ebi.ac.uk/pub/databases/alphafold/latest/UP000024404_6282_ONCVO_v4.tar) |
| *Oryza sativa* | [UP000059680](https://www.uniprot.org/proteomes/UP000059680) | 43,649 | [4,516 MB](https://ftp.ebi.ac.uk/pub/databases/alphafold/latest/UP000059680_39947_ORYSJ_v4.tar) |
| *Paracoccidioides lutzii* | [UP000002059](https://www.uniprot.org/proteomes/UP000002059) | 8,794 | [1,298 MB](https://ftp.ebi.ac.uk/pub/databases/alphafold/latest/UP000002059_502779_PARBA_v4.tar) |
| *Plasmodium falciparum* | [UP000001450](https://www.uniprot.org/proteomes/UP000001450) | 5,187 | [1,152 MB](https://ftp.ebi.ac.uk/pub/databases/alphafold/latest/UP000001450_36329_PLAF7_v4.tar) |
| *Pseudomonas aeruginosa* | [UP000002438](https://www.uniprot.org/proteomes/UP000002438) | 5,556 | [615 MB](https://ftp.ebi.ac.uk/pub/databases/alphafold/latest/UP000002438_208964_PSEAE_v4.tar) |
| *Rattus norvegicus* | [UP000002494](https://www.uniprot.org/proteomes/UP000002494) | 21,270 | [3,473 MB](https://ftp.ebi.ac.uk/pub/databases/alphafold/latest/UP000002494_10116_RAT_v4.tar) |
| *Saccharomyces cerevisiae* | [UP000002311](https://www.uniprot.org/proteomes/UP000002311) | 6,039 | [979 MB](https://ftp.ebi.ac.uk/pub/databases/alphafold/latest/UP000002311_559292_YEAST_v4.tar) |
| *Salmonella typhimurium* | [UP000001014](https://www.uniprot.org/proteomes/UP000001014) | 4,526 | [479 MB](https://ftp.ebi.ac.uk/pub/databases/alphafold/latest/UP000001014_99287_SALTY_v4.tar) |
| *Schistosoma mansoni* | [UP000008854](https://www.uniprot.org/proteomes/UP000008854) | 13,865 | [2,549 MB](https://ftp.ebi.ac.uk/pub/databases/alphafold/latest/UP000008854_6183_SCHMA_v4.tar) |
| *Schizosaccharomyces pombe* | [UP000002485](https://www.uniprot.org/proteomes/UP000002485) | 5,128 | [792 MB](https://ftp.ebi.ac.uk/pub/databases/alphafold/latest/UP000002485_284812_SCHPO_v4.tar) |
| *Shigella dysenteriae* | [UP000002716](https://www.uniprot.org/proteomes/UP000002716) | 3,893 | [375 MB](https://ftp.ebi.ac.uk/pub/databases/alphafold/latest/UP000002716_300267_SHIDS_v4.tar) |
| *Sporothrix schenckii* | [UP000018087](https://www.uniprot.org/proteomes/UP000018087) | 8,652 | [1,523 MB](https://ftp.ebi.ac.uk/pub/databases/alphafold/latest/UP000018087_1391915_SPOS1_v4.tar) |
| *Staphylococcus aureus* | [UP000008816](https://www.uniprot.org/proteomes/UP000008816) | 2,888 | [275 MB](https://ftp.ebi.ac.uk/pub/databases/alphafold/latest/UP000008816_93061_STAA8_v4.tar) |
| *Streptococcus pneumoniae* | [UP000000586](https://www.uniprot.org/proteomes/UP000000586) | 2,030 | [203 MB](https://ftp.ebi.ac.uk/pub/databases/alphafold/latest/UP000000586_171101_STRR6_v4.tar) |
| *Strongyloides stercoralis* | [UP000035681](https://www.uniprot.org/proteomes/UP000035681) | 12,613 | [1,898 MB](https://ftp.ebi.ac.uk/pub/databases/alphafold/latest/UP000035681_6248_STRER_v4.tar) |
| *Trichuris trichiura* | [UP000030665](https://www.uniprot.org/proteomes/UP000030665) | 9,564 | [1,365 MB](https://ftp.ebi.ac.uk/pub/databases/alphafold/latest/UP000030665_36087_TRITR_v4.tar) |
| *Trypanosoma brucei* | [UP000008524](https://www.uniprot.org/proteomes/UP000008524) | 8,491 | [1,348 MB](https://ftp.ebi.ac.uk/pub/databases/alphafold/latest/UP000008524_185431_TRYB2_v4.tar) |
| *Trypanosoma cruzi* | [UP000002296](https://www.uniprot.org/proteomes/UP000002296) | 19,036 | [2,965 MB](https://ftp.ebi.ac.uk/pub/databases/alphafold/latest/UP000002296_353153_TRYCC_v4.tar) |
| *Wuchereria bancrofti* | [UP000270924](https://www.uniprot.org/proteomes/UP000270924) | 12,721 | [1,420 MB](https://ftp.ebi.ac.uk/pub/databases/alphafold/latest/UP000270924_6293_WUCBA_v4.tar) |
| *Zea mays* | [UP000007305](https://www.uniprot.org/proteomes/UP000007305) | 39,299 | [5,122 MB](https://ftp.ebi.ac.uk/pub/databases/alphafold/latest/UP000007305_4577_MAIZE_v4.tar) |

**Supplementary Table 1 - Proteomes available for download in bulk from FTP**
